# Monkeypox Case Investigation — Cook County Jail, Chicago, Illinois, July–August 2022

**DOI:** 10.15585/mmwr.mm7140e2

**Published:** 2022-10-07

**Authors:** Liesl M. Hagan, Amy Beeson, Sarah Hughes, Rashida Hassan, Lauren Tietje, Ashley A. Meehan, Hillary Spencer, Janice Turner, Morgan Richardson, Jourdan Howard, Anne Schultz, Salma Ali, Margaret Mary Butler, Diana Arce Garza, Clint N. Morgan, Chantal Kling, Nicolle Baird, Michael B. Townsend, William C. Carson, David Lowe, Nhien T. Wynn, Stephanie R. Black, Janna L. Kerins, Josh Rafinski, Andrew Defuniak, Priscilla Auguston, Emily Mosites, Isaac Ghinai, Chad Zawitz

**Affiliations:** ^1^CDC Monkeypox Emergency Response Team; ^2^Epidemic Intelligence Service, CDC; ^3^Chicago Department of Public Health, Chicago, Illinois; ^4^Rush University Medical Center, Chicago, Illinois; ^5^University of Illinois at Chicago, Chicago, Illinois; ^6^Division of High-Consequence Pathogens and Pathology, National Center for Emerging and Zoonotic Infectious Diseases, CDC; ^7^Cermak Health Services of Cook County, Chicago, Illinois; ^8^Cook County Health, Chicago, Illinois.

Knowledge about monkeypox transmission risk in congregate settings is limited. In July 2022, the Chicago Department of Public Health (CDPH) confirmed a case of monkeypox in a person detained in Cook County Jail (CCJ) in Chicago, Illinois. This case was the first identified in a correctional setting in the United States and reported to CDC during the 2022 multinational monkeypox outbreak. CDPH collaborated with CCJ, the Illinois Department of Public Health (IDPH), and CDC to evaluate transmission risk within the facility. Fifty-seven residents were classified as having intermediate-risk exposures to the patient with monkeypox during the 7-day interval between the patient’s symptom onset and his isolation. (Intermediate-risk exposure was defined as potentially being within 6 ft of the patient with monkeypox for a total of ≥3 hours cumulatively, without wearing a surgical mask or respirator, or potentially having contact between their own intact skin or clothing and the skin lesions or body fluids from the patient or with materials that were in contact with the patient’s skin lesions or body fluids.) No secondary cases were identified among a subset of 62% of these potentially exposed residents who received symptom monitoring, serologic testing, or both. Thirteen residents accepted postexposure prophylaxis (PEP), with higher acceptance among those who were offered counseling individually or in small groups than among those who were offered PEP together in a large group. *Monkeypox virus* (MPXV) DNA, but no viable virus, was detected on one surface in a dormitory where the patient had been housed with other residents before he was isolated. Although monkeypox transmission might be limited in similar congregate settings in the absence of higher-risk exposures, congregate facilities should maintain recommended infection control practices in response to monkeypox cases, including placing the person with monkeypox in medical isolation and promptly and thoroughly cleaning and disinfecting spaces where the person has spent time. In addition, officials should provide information to residents and staff members about monkeypox symptoms and transmission modes, facilitate confidential monkeypox risk and symptom disclosure and prompt medical evaluation for symptoms that are reported, and provide PEP counseling in a private setting.

## Investigation and Results

CCJ houses approximately 6,000 residents in cell-based and dormitory-based units across 16 buildings. The monkeypox case occurred in a resident who was booked into jail in mid-July 2022 (investigation day 1) and assigned to two congregate dormitories used for intake (dormitories A and B)^†^ during the 7 days preceding his isolation for suspected monkeypox. On day 7, the resident placed a written request for health services, reporting swollen genitals, and CCJ health care personnel ordered a sexually transmitted infection laboratory panel. On day 8, health care administrators received a call from one of the patient’s family members alerting them to the possibility that the patient might have monkeypox; he was then evaluated in person and isolated. Lesion swab specimens collected for nonvariola *Orthopoxvirus* (NVO) testing on day 9 returned a positive result on day 11. During evaluation, the patient reported first noticing a localized rash on day 2, which subsequently spread over much of his body and was accompanied by fatigue and body aches before he was isolated. IDPH requested a CDC deployment team to assist with the investigation. This activity was reviewed and approved by CDC and conducted consistent with applicable federal law and CDC policy.^§^

Fifty-seven other residents were housed with the patient for 1–7 nights (median = 5 nights) before he was isolated (Table) (Figure). Although CCJ policy required indoor mask use as a COVID-19 prevention strategy during the period of this investigation, enforcing mask use 24 hours per day in correctional facilities is challenging and mask usage is often low; the patient and other residents were not observed wearing masks consistently during this time. The patient reported during an interview that he had had no skin-to-skin or sexual contact with other residents, and no such contact between the patient and other residents was observed during review of security video footage. Because of the difficulty in ascertaining whether each resident sharing a dormitory with the patient met criteria for intermediate-risk exposure versus lower-risk exposure (simply entering the living space of a person with monkeypox), all 57 residents were conservatively categorized as having had intermediate-risk exposure.^¶^

**TABLE:** Characteristics of residents potentially exposed to *Monkeypox virus* and who participated in elements of a field investigation (N = 57) — Cook County Jail, Chicago, Illinois, July–August 2022

Characteristic	No. (%)				
	Potentially exposed*	Offered PEP^†^	Accepted PEP^†^	Accepted testing	Individually interviewed
**Total**	**57**	**36**	**13**	**14**	**16**
**Age, yrs, median (range)**	38 (21–63)	38 (21–62)	33 (22–58)	38 (21–62)	43 (21–62)
**No. of nights potentially exposed, median (range)**	5 (1–7)	5 (1–7)	5 (1–6)	5 (1–7)	5 (1–7)
**Sex**					
Male	57 (100)	36 (100)	13 (100)	14 (100)	16 (100)
**Race or ethnicity**					
Black or African American, non-Hispanic	30 (53)	18 (50)	4 (31)	7 (50)	9 (56)
White or Caucasian, non-Hispanic	17 (30)	12 (33)	4 (31)	4 (29)	4 (25)
Hispanic or Latino	8 (14)	5 (14)	4 (31)	2 (14)	2 (13)
Asian, non-Hispanic	2 (4)	1 (3)	1 (8)	1 (7)	1 (6)

**Abbreviation**: PEP = postexposure prophylaxis.

* Classified as having intermediate-risk exposure to the patient with monkeypox (i.e., were potentially within 6 ft of the patient for a cumulative period of ≥3 hours without wearing a surgical mask or respirator, or potentially had contact between their own intact skin or clothing and the skin lesions or body fluids from the patient with monkeypox or with materials that were in contact with the patient’s skin lesions or body fluids)**.**

**^†^** JYNNEOS vaccine.

**FIGURE:**
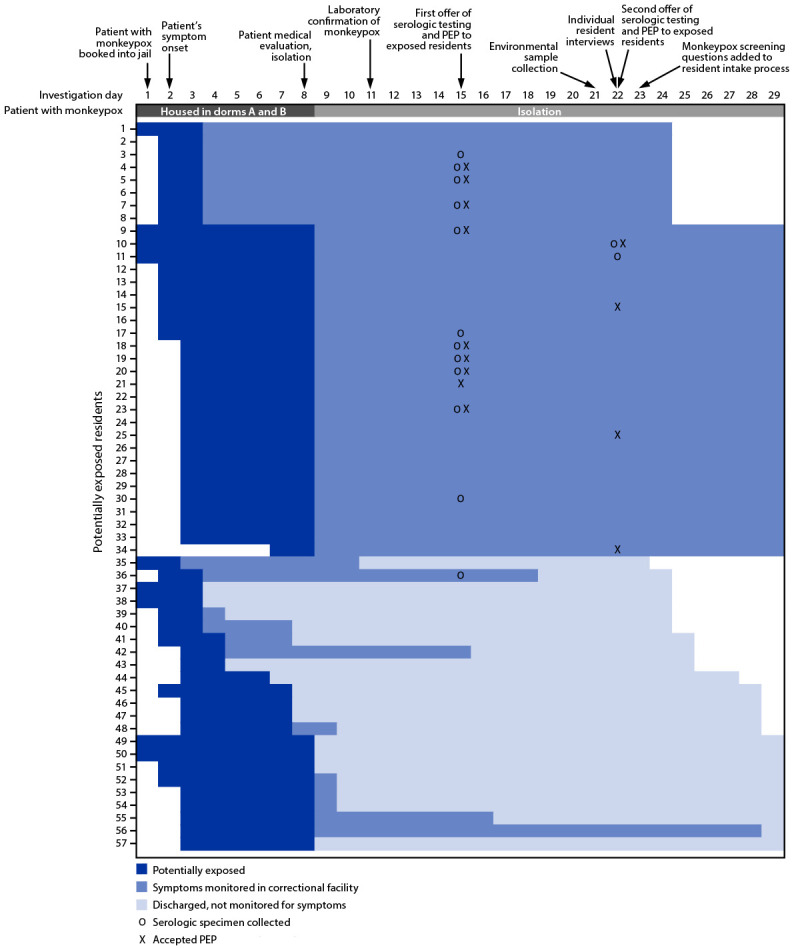
Follow-up of 57 residents potentially exposed to *Monkeypox virus* — Cook County Jail, Chicago, Illinois, July–August 2022 **Abbreviation:** PEP = postexposure prophylaxis.

On investigation day 15, serologic testing was offered to the 36 potentially exposed residents who were still in detention. One week later, on investigation day 22, serologic testing was again offered to those who had declined the first offer. Among all 36 residents still in detention, a total of 14 (39%) consented to testing. Specimens were tested by enzyme-linked immunosorbent assay for anti-*Orthopoxvirus* immunoglobulin (Ig) M (a transient marker of acute infection or recent vaccination) and IgG (a long-lived marker generated during infection or vaccination) (*1*). None of the specimens tested positive for IgM. Specimens from three residents tested positive for IgG; all three were old enough to have received routine childhood smallpox vaccination, although their previous smallpox vaccination history could not be confirmed.**

On investigation day 8, after the patient was isolated, CCJ resident-workers cleaned and disinfected dormitories A and B. To evaluate the extent of remaining surface contamination, 54 environmental samples were collected from both dormitories on investigation day 21, which was 18 days after the patient had been in dormitory A and 13 days after he had been in dormitory B.^††^ One dormitory B sample, collected from a vertical, painted concrete slab at the head of the patient’s bed, tested positive for NVO DNA by real-time polymerase chain reaction (PCR) and was confirmed by Clade II MPXV-specific PCR; viral culture was negative^§§^ (*2*).

To identify possible exposure patterns in the dormitories and to assess residents’ knowledge about monkeypox, 16 potentially exposed residents were interviewed individually.^¶¶^ The majority of residents (12) reported washing their clothes in communal showers or sinks in the dormitory. Some residents reported sharing personal hygiene items (five) or eating utensils (four) with other residents, engaging in physical altercations (four), sitting on other residents’ beds (three), or sharing or touching other residents’ linens (two). None reported sexual contact with others while in CCJ. The majority (13) also reported hearing about monkeypox for the first time while detained in CCJ. Residents’ knowledge about monkeypox symptoms, transmission modes, and exposure risks varied but was generally low.

## Public Health Response

CDPH recommended PEP and daily symptom monitoring for 21 days after last exposure for all 57 potentially exposed residents.*** CCJ notified the residents of their potential exposure and, out of an abundance of caution, placed them under quarantine precautions within dormitories A and B. One resident reported a rash on investigation day 22 and was evaluated the same day; test results for NVO were negative. Among the 57 residents, 35 (61%) remained in detention for their full 21-day monitoring period (33 in CCJ and two transferred to a state prison). The remaining 22 (39%) residents were discharged to the community before conclusion of their 21-day monitoring period and were lost to follow-up. However, CDPH cross-checked the names of the discharged residents with the Illinois state testing database and confirmed that none had a record of monkeypox testing in the 30 days after their last potential exposure in CCJ.^†††^

On investigation day 15, PEP with JYNNEOS vaccine was offered to the 36 (63%) potentially exposed residents who were still in detention at that time (the same 36 residents who were also offered serologic testing on day 15 as part of the investigation). Dormitory B residents received PEP information as a large group, followed by a public roll call offering PEP to each resident. Staff members reported difficulty communicating effectively in the large group; only three of 25 (12%) residents who were offered PEP in this setting accepted. In subsequent individual interviews, several dormitory B residents indicated they did not want to receive the vaccine in front of others, did not know enough about the vaccine or potential side effects, or thought they were being offered a COVID-19 vaccine. In contrast, dormitory A residents were escorted to a separate room individually or in groups of two, where they were counseled and offered PEP; six of 11 offered PEP in this setting accepted. On day 22, PEP was reoffered individually to eight residents from dormitories A and B participating in individual interviews who had declined the first PEP offer; four accepted. Overall, 13 (23%) of 57 residents received PEP 7–14 days after their last potential exposure (median = 12 days).

In early August, CCJ added monkeypox screening questions (presence of rash or known close contact with someone with monkeypox) to the intake process for new residents entering CCJ. Shortly thereafter, a newly detained resident answered “no” to all screening questions but later, in a private exam room with a medical provider, disclosed that he had been hospitalized with monkeypox 2 weeks before his arrest.

## Discussion

After a CCJ resident with symptomatic monkeypox spent 7 days in congregate housing, no additional cases were detected among a subset of residents classified as having intermediate-risk exposures (62%) who were monitored for symptoms or who received serologic testing. Although the patient reported no skin-to-skin or sexual contact with other residents, all residents slept in the same room with the patient and shared living and dining spaces and bathroom facilities. These findings suggest that monkeypox transmission might be limited in similar congregate settings in the absence of higher-risk exposures such as skin-to-skin or sexual contact (the primary transmission modes identified during the current multinational outbreak). Current CDC guidance does not recommend quarantine for exposed persons who remain asymptomatic; these findings affirm application of this guidance within congregate settings.^§§§^

Although this investigation found no evidence of skin-to-skin or sexual contact among residents in CCJ, previous research emphasizes that persons who are incarcerated might not disclose intimate or sexual contact within the facility because of potential stigma, retaliation, or disciplinary consequences (*3*). Furthermore, monkeypox transmission has been documented in correctional settings previously, including a cluster of five cases and an outbreak of 21 cases in Nigerian prisons in 2017 and 2022, respectively, where the transmission modes could not be definitively ascertained (*4*,*5*). In this investigation, some residents disclosed contact patterns in the dormitory overall (not necessarily with the patient with monkeypox) that have previously been associated with transmission in household studies (e.g., sharing eating utensils and linens) (*6*). Thus, correctional facilities need to remain vigilant for potential cases of monkeypox while transmission continues to occur in the United States. 

Results of PCR testing of surfaces in the shared CCJ dormitories indicate that at least one surface retained MPXV DNA at the time of sampling: a vertical, painted concrete slab at the head of the patient’s bed. Residents commonly lean against this type of surface while sitting in bed, or drape damp clothing and towels over it to dry. Although no viable virus was detected on the surface at the time of sampling, studies with vaccinia virus have found viable virus persisting up to 28 days on a similar surface, indicating the importance of thoroughly disinfecting all areas where a person with monkeypox has spent time, including all surfaces they might have touched or that might have had contact with their clothing or linens (*7*). Facilities should ensure that residents and staff members responsible for cleaning and disinfection receive adequate training, supplies, and oversight to complete these tasks.

Approximately one third of CCJ residents who were exposed to the patient with monkeypox were discharged before PEP was offered, and those who accepted PEP received it 7–14 days after exposure, outside the 4-day window recommended to prevent infection. Among residents offered PEP, approximately one third accepted it, a rate lower than that reported among community and health care contacts during previous monkeypox outbreaks (*8*). Notably, PEP acceptance was higher among residents who received individual or small group counseling (55%) than among those who were offered PEP while in a large group (12%). Similarly, a resident booked into CCJ after the conclusion of this investigation privately disclosed a recent hospitalization for monkeypox after previously answering “no” to all screening questions asked in a semipublic intake space.

The findings in this report are subject to at least five limitations. First, exposure risk assessment was challenging in the congregate housing setting, and some residents classified as having intermediate-risk exposure actually could have had a lower-risk exposure. Second, serologic testing and symptom monitoring were completed for only 25% and 62% of exposed residents, respectively. Third, serologic testing was performed 7 days after potential exposure for some residents, when they might not yet have seroconverted, possibly resulting in misclassification of secondary cases. Fourth, monkeypox-related stigma or desire to avoid isolation could have limited self-report of symptoms or higher-risk contact such as sexual activity. Finally, findings might not be generalizable to all congregate settings because of variation in facility layout, ventilation, housing density, laundry practices, and adherence to infection prevention and control protocols, and because of differences in viral shedding and infectious period among persons with monkeypox. Additional data can further elucidate transmission risk in congregate settings overall.

Correctional facilities can reduce monkeypox transmission risk by following public health recommendations (Box). First, facilities should maintain infection control protocols in response to cases, including isolation of persons with suspected monkeypox and prompt and thorough cleaning and disinfection of all areas where the person has spent time^¶¶¶^ (*9*). Second, facilities should provide monkeypox prevention information to residents and staff members, including information about avoiding sexual contact in the custody setting and avoiding common practices such as sharing eating utensils and linens. Third, facility officials should follow health department guidance for postexposure symptom monitoring and PEP, provide information about monkeypox signs and symptoms and how to report them confidentially, and ensure prompt evaluation when residents do report symptoms. Using private spaces during intake screening, exposure notification, and PEP counseling can support disclosure of sensitive information and could improve acceptance of public health recommendations.

BOX **Public health messages related to monkeypox prevention in correctional settings — United States, 2022**

**When monkeypox is suspected, promptly isolate the affected person, evaluate them for testing, and alert the health department for further support and guidance (https://www.cdc.gov/poxvirus/monkeypox/clinicians/clinical-recognition.html).**

- Multiple residents with confirmed monkeypox can be housed together.
- Persons entering the isolation space or handling laundry from persons with monkeypox should wear recommended PPE (https://www.cdc.gov/poxvirus/monkeypox/community/congregate.html). Patients should wear a mask and cover lesions if they leave the isolation space.
- Collect and contain soiled laundry and linens from a person with monkeypox in a container that can be disinfected, or a laundry bag that can be laundered along with the soiled items from the person with monkeypox. Do not shake or handle laundry in a manner that might disperse infectious material. Launder separately from other residents’ laundry using regular detergent.
- Support patients’ mental health during isolation, and ensure they have regular access to showers, hygiene supplies, and clean clothing and linens.

**Surface contamination with *Monkeypox virus* can occur and can contribute to transmission.**

- Ensure prompt and thorough cleaning and disinfection in spaces where a person with monkeypox has spent time. Include all surfaces that someone with monkeypox might have touched or places they might have stored or placed soiled clothing, towels, or linens.
- Perform disinfection using an EPA-registered disinfectant with an Emerging Viral Pathogens claim (https://www.epa.gov/coronavirus/what-emerging-viral-pathogen-claim) found on EPA’s List Q (https://www.epa.gov/pesticide-registration/disinfectants-emerging-viral-pathogens-evps-list-q), according to instructions on the product label.
- Provide sufficient training, supplies, oversight, and PPE (https://www.cdc.gov/poxvirus/monkeypox/clinicians/clinical-recognition.html) to residents and staff members responsible for cleaning and disinfection. For more information, see Workplace Solutions: Safe and Proper Use of Disinfectants to Reduce Viral Surface Contamination in Correctional Facilities (https://www.cdc.gov/niosh/docs/wp-solutions/2021-121/).

**Contact tracing can help to prevent further transmission. However, persons exposed to *Monkeypox virus* do not need to quarantine if they do not have signs or symptoms consistent with monkeypox. Confidentially inform residents and staff members of potential exposure to prevent stigma and to encourage disclosure of high-risk contact that might have occurred (https://www.cdc.gov/poxvirus/monkeypox/clinicians/monitoring.html).**

**Follow health department guidance for symptom monitoring and PEP for residents and staff members who have been exposed. Ensure that residents and staff members know the symptoms of monkeypox and ensure that residents can confidentially report symptoms or contact with a person with monkeypox. Evaluate residents promptly when they report symptoms.**

**When indicated, offer PEP as soon as possible after exposure to prevent loss to follow-up (especially in high-throughput settings like jails) and to best prevent infection.**

- For maximum effectiveness, PEP should be offered within 4 days of exposure.
- Discuss options with the health department for offering PrEP vaccination to residents who might be at increased risk for monkeypox in the facility or after release (https://www.cdc.gov/poxvirus/monkeypox/vaccines/vaccine-basics.html).
- PEP acceptability might be lower in some correctional environments than in other settings and can be supported through individual, confidential discussions between residents and trusted communicators, such as medical providers.

**Facilitate disclosure of a known monkeypox diagnosis, symptoms, or risk factors at intake by asking screening questions in private spaces.**

**Residents and staff members might not have adequate information about monkeypox or adequate access to hygiene and cleaning supplies to protect themselves. Provide correctional facility residents with no-cost supplies to enable them to wash their hands and clean their living areas frequently and provide information about avoiding skin-to-skin and sexual contact in custody settings and avoiding common interactions that could lead to exposure, such as sharing personal items, clothing, linens, eating utensils, cups, and bowls.**

**Abbreviations:** EPA = Environmental Protection Agency; PEP = postexposure prophylaxis; PPE = personal protective equipment; PrEP = preexposure prophylaxis.

**Summary**

**What is already known about this topic?**

Knowledge about monkeypox transmission risk in congregate settings is limited.

**What is added by this report?**

After a jail resident with symptomatic monkeypox spent 7 days in congregate housing, no cases were detected among a subset of residents with intermediate-risk exposures (being within 6 ft of the patient for ≥3 hours without wearing a mask) who received symptom monitoring or serologic testing. *Monkeypox virus* DNA, but no viable virus, was detected on one surface. Postexposure prophylaxis (PEP) acceptance was highest when offered privately.

**What are the implications for public health practice?**

Although monkeypox transmission might be limited in similar congregate settings without higher-risk exposures, facilities should implement recommended infection control practices and provide prevention education including confidential PEP counseling.

## Footnotes

† Dormitories A and B are each 2,950 ft^2^ with 10.5-ft high ceilings and a congregate bathroom with five combination toilet-sink units and five shower stalls. Each has 39 fixed single bed platforms spaced a minimum of 3 ft apart. During the investigation period, the ventilation system typically provided five to six air changes per hour for both dormitories; when outdoor air temperature was >90°F (>32°C) for brief periods on 2 days, three to four air changes per hour occurred. During the period when the patient with monkeypox was housed in dormitories A and B, residents were served meals at communal tables inside the dormitories and spent an average of 20–24 hrs per day in the dormitories.

§ 45 C.F.R. part 46, 21 C.F.R. part 56; 42 U.S.C. Sect. 241(d); 5 U.S.C. Sect. 552a; 44 U.S.C. Sect. 3501 et seq.

¶
https://www.cdc.gov/poxvirus/monkeypox/clinicians/monitoring.html

** One resident had an equivocal IgM result 7 days after last potential exposure to the patient but reported no symptoms at the time of specimen collection or during his 21-day incubation period. Because he received PEP, repeat serologic testing was not performed; seroconversion in progress cannot be definitively ruled out.

†† Surfaces included painted concrete bed platforms, desks, and stools in the spaces assigned to the patient before he was isolated, as well as metal toilet rims and flush buttons, metal sink and shower buttons, and plastic telephone receivers shared in communal spaces.

§§ Polyester-tipped applicator swabs were prewetted with phosphate buffered saline (PBS) solution before swabbing environmental surfaces, then stored and transported in 300 *μ*L of PBS solution and kept frozen until processing using the swab extraction tube system (Roche); DNA was extracted from the swab eluate before PCR testing.

¶¶ Residents were asked about their monkeypox knowledge (including transmission modes, prevention, and symptoms) and the types of contact they had with other persons in the jail or with shared objects. The identity of the patient with monkeypox was not disclosed; thus, questions about contact with others in the jail did not specify whether it occurred with the patient versus with other persons in the dormitory.

*** Nursing staff members checked vital signs and asked residents about symptoms once daily. During days 11–13, monitoring included symptoms of influenza-like illness and COVID-19; on day 15, new-onset rash was added. Staff members who worked in dormitories A or B were determined to have lower-risk exposures and were not offered PEP or advised to self-monitor for symptoms.

††† Telephone numbers were available for 17 of the 22 potentially exposed residents discharged to the community before their 21-day monitoring period ended. CDPH staff members called all 17 numbers but were unable to reach any of the discharged residents to ask about monkeypox symptoms they might have experienced during the monitoring period.

§§§ https://www.cdc.gov/poxvirus/monkeypox/clinicians/monitoring.html; https://www.cdc.gov/poxvirus/monkeypox/community/congregate.htm

¶¶¶ Using an Environmental Protection Agency (EPA)–registered disinfectant with an Emerging Viral Pathogens claim (https://www.epa.gov/coronavirus/what-emerging-viral-pathogen-claim) found on EPA’s List Q (https://www.epa.gov/pesticide-registration/disinfectants-emerging-viral-pathogens-evps-list-q).
